# First-principles calculations steered multi-task transformer model to screen dual-atom catalysts for C-H activation

**DOI:** 10.1016/j.isci.2025.114182

**Published:** 2025-11-21

**Authors:** BaiRan Wang, WeiHang Xu, XiaoYing Sun, Qi Ji, LingLing Shang, Zhen Zhao, Bo Li

**Affiliations:** 1 Institute of Catalysis for Energy and Environment, College of Chemistry and Chemical Engineering, Shenyang Normal University, Shenyang 110034, China; 2 State Key Laboratory of Heavy Oil Processing, China University of Petroleum, Beijing 102249, China

**Keywords:** Chemical reaction, Catalysis, Molecular modeling, Computational chemistry

## Abstract

Activating C–H bonds in light alkanes (methane, ethane, and propane) remains a major challenge in heterogeneous catalysis due to their high stability, while dual-atom catalysts (DACs) offer a promising balance of activity and durability. Herein, we developed an optimized multi-task transformer model to accelerate active DACs design by integrating first-principles calculations with machine learning. Over 200 DACs composed of fourth/fifth period transition metals anchored on N-doped graphene were studied. The transformer, modified with additional decoder layers and attention head, outperformed conventional methods (K-nearest neighbors, resource description framework, and gradient boosting regression) for small datasets, achieving R^2^ > 0.85 in predicting both adsorption energies and C-H activation barriers. Gradient boosting regression tree analysis identified the metal-N coordination distance as the key performance-determining factor. This work provides an accurate predictive model for efficient DACs screening in light alkane conversion and advances DACs design principles.

## Highlights

• Optimized the multi-task transformer model for small dataset prediction
• The metal nitrogen distance was the key factor for design of DACs
• Simultaneous prediction of adsorption and activation enable a balanced estimation

## Graphical abstract

**Figure:**
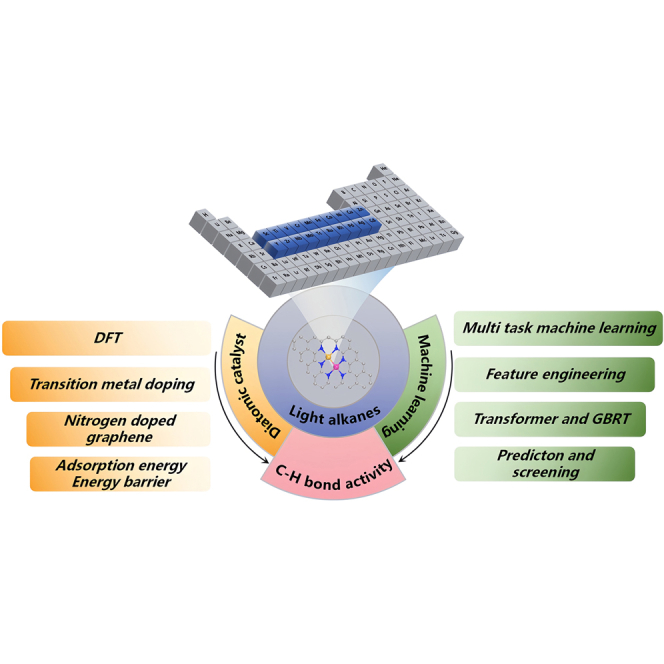

## Introduction

Light alkane molecules such as methane, ethane, and propane are acyclic saturated hydrocarbons and the main components of natural gas and crude oil. Although light alkanes are very attractive raw material for many high value-added products such as alcohols, alkanes, and alkenes, their activation is extremely difficult due to their stable C–H bonds, negligible electron affinity, high ionization energy, and low polarizability.^1^ For example, the bond dissociation energy of methane is 439 kJ/mol^2^ which makes its activation at ambient condition extremely challenging, and it is coined as “holy grail” problem in catalysis.^3^ On the other hand, the activation of the first C–H bond in light alkanes is generally considered as the rate controlling step of the relevant catalytic process,^4^^,^^5^and the reactivity is significantly increased resulted from broken symmetry after first C–H bond broken.

The commonly used catalysts for the thermal catalytic conversion of light alkanes include metal catalysts,^6^ metal oxide catalysts,^7^ composite catalysts,^8^ and molecular sieves.^9^ Although the achievement of light alkanes catalytic activation is substantial, there is still lots of room for further improvements considering efficiency and sustainability. In recent years, the concept of single atom catalysts (SACs) caused a storm in heterogeneous catalysis due to its promise of excellent activity and selectivity together with high metal atom utility efficiency.^10^ As shown in our previous work of SAC in propane activation,^11^ single Pt anchored on doped graphene or boron nitride can effectively break C–H bond in propane molecule, and more importantly the variation of coordinated support atoms can tune the catalytic properties of SAC in a controlled manner. Although SACs indeed showed great potential in light alkane activation, it often suffered from rigidity of scaling relation resulted from single type active species which limited its optimization strategy and further improvements. Recently the rising of dual-atom catalysts (DACs) provides an alternative way to circumvent the bottleneck issues of single atom catalysis while retaining some advantages of SAC such as high atom efficiency and selectivity.^12^ The two atomic active centers, either homonuclear or heteronuclear configurations, in principle can provide more room for optimization by a flexible combination of metal atoms to tune binding energy of adsorbates for instance, and the possible synergistic effect between two active spices could further enhance the catalytic performance.^13^

On the other hand, DACs has exhibited great potential in light alkane activation. Chai et al. synthesized DACs of Zn_1_Co_1_ anchored on nitrogen-doped carbon and applied in propane dehydrogenation. They found that DACs of Zn_1_Co_1_ delivered two and three times TOF (Turnover Frequency) over the counterparts of single Zn and Co, respectively, and attributed this improvement to the reduced barrier of C–H bond activation in propane which is 126, 141, and 170 kJ/mol for Zn_1_Co_1_, Zn_1_, and Co_1_, respectively.^14^ Therefore, it is clearly indicated that there existed synergistic effects between Zn and Co which enable DACs to outperform the SAC, and this synergistic effect is identified as the pronounced contributions around Fermi level from coordinated nitrogen caused by the interaction between Zn and Co. More interestingly, DACs not only showed improved performance over SAC but also provide alternative reaction pathway which is also different from SAC. Liu et al. prepared FeCo-DACs catalyst anchored on nitrogen-doped carbon,^15^ and applied in alcohol dehydrogenation which showed 70% product yields while the yields of Fe and Co SACs are only 22% and 19%, respectively, under same experimental condition. It is noted that alcohol dehydrogenation catalyzed by FeCo DACs catalyst proceeded via O–H bond activation as first step on pathway, and it becomes to be C–H bond activation for SACs catalysts. Hence, DACs showed different features in term of both performance and mechanism compared with SACs. Overall, DACs indeed appeared to be a new class of catalysts for C–H bond activation in light alkanes other than SACs.

Considering the promise of DAC catalyst in catalytic light alkane activation, it is urgent to establish an effective model for rational design to replace the conventional trial-error method. In recent years, with the rapid developments of machine learning (ML) methods, the combination between first principles calculations and ML method became a win-win way to tackle the design principle for catalysts.^16^ As shown in our previous work,^17^ DFT (density functional theory) calculated adsorption energy and reaction barrier as training data for supervised ML methods including RFR (random forest regression), KNR (K-Neighbors Regressor), ABR (adaptive bitrate), and GBR (gradient boosting regression) successfully identified the most effective promoter for Pt catalyst in propane activation. Moreover, equations derived from SISSO (Sure Independence Screening and Sparsifying Operator) method are obtained to predict the barrier of C–H bond activation of both propane and propene which provided a quantitative description on PDH (propane dehydrogenation) reactivity and selectivity. Zhao et al. carried out a combined study of experimental, DFT and 6 to examine Rh-based cluster reactivity toward to C–H bond activation of light C_1_-C_4_ alkanes.^18^ DFT calculations explored the configuration space of cluster and identified the favorable structures, while experimental measurements provided rate constant of C–H bond activation as dataset. Four popular ML methods included RFR, SVR (support vector regression), GBRT (gradient boosting regression tree), and BPANN (back propagation neural network) are employed to yield a quantitively predictive model and also identified the most important features for C–H bond activation. In the study of screening DACs for HER (hydrogen evolution reaction), the graph convolution neural network method was used to automatically capture the topology, local atomic environment, and atomic properties of nitrogen-doped graphene-based DACs, and accurately predict the Gibbs free energy of hydrogen adsorption of 435 DACs. The RMSE (Root Mean Square Error) of the test set was only 0.27 eV^19^ which pave way to find optimal HER catalysts. In another study, the random forest algorithm was used to analyze the stability of DACs anchored on nitrogen-doped graphene with intrinsic property. By constructing a model containing 26 intrinsic descriptors such as electronegativity, atomic number, and d electron number, 465 DACs were evaluated and a few candidates are identified as effective CO_2_RR catalysts with low overpotential by breaking scaling relation; moreover the R^2^ of the training set and the test set reached 0.94 and 0.97, respectively, and the electronegativity was the key factor affecting the stability.^20^ Zheng et al. used the transformer algorithm as the core feature engineering tool, generated composite features and reduced dimensions through genetic programming, and helped to efficiently screen high-performance HER/DER (deuterium evolution reaction) catalysts such as NbSi_2_N_4_ and VSi_2_N_4_ from 276 MA_2_Z_4_ materials, with balancing the prediction accuracy and calculation cost of ML model.^21^

In current work, the methodology of ML and first-principles calculations is further extended to explore the active DACs anchored on nitrogen-doped graphene for C–H bond activation of light alkane molecules including methane, ethane, and propane. Different from previous studies,^22^^,^^23^ a multitask transformer model is employed to simultaneously predict both adsorption energy and C–H bond activation barrier of light alkane molecules which will give an unbiased evaluation of reactivity of DACs as both factors are crucial for the catalytic performance. Also, the most influential feature associated with performance is determined for DACs and is useful to uncover the underlying principle for catalyst design. The careful curated model from current work provides an effective way to screen active DACs for light alkane molecule activation.

## Results and discussion

### First principles calculations

**Figure 1:**
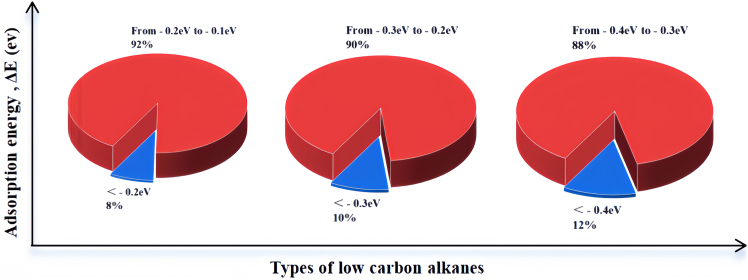
The category of DFT calculated adsorption energy for methane, ethane, and propane (from left to right)

First of all, the formation energy of DACs candidates used in ML regression are obtained as shown in Figure S2 to determine the stability which indicated that DACs formation is an exothermic process. The adsorption of alkanes molecule is a useful indicator for catalyst activity as it often reported that partial pressure of light alkane molecule is positively correlated with the conversion.^24^ Hence the strong adsorption is often deemed as beneficial factor for C–H bond activation. As expected, the adsorption of light alkane molecule is a kind of weak physisorption which is below −0.5 eV. As shown in Figure 1, the adsorption energy of majority methane, ethane, and propane molecules is in the range of −0.1 to −0.2, −0.2 to −0.3, and −0.3 to −0.4 eV, respectively, which also showed a gradual increase with increasing carbon atoms in alkane molecules. As shown in Figure S3, one of hydrogen atoms in adsorbed alkane molecule is pointing to metal site of DACs and the typical height of adsorbed light alkane molecule is around 2.1 Å which further demonstrated the nature of physisorption.

**Figure 2:**
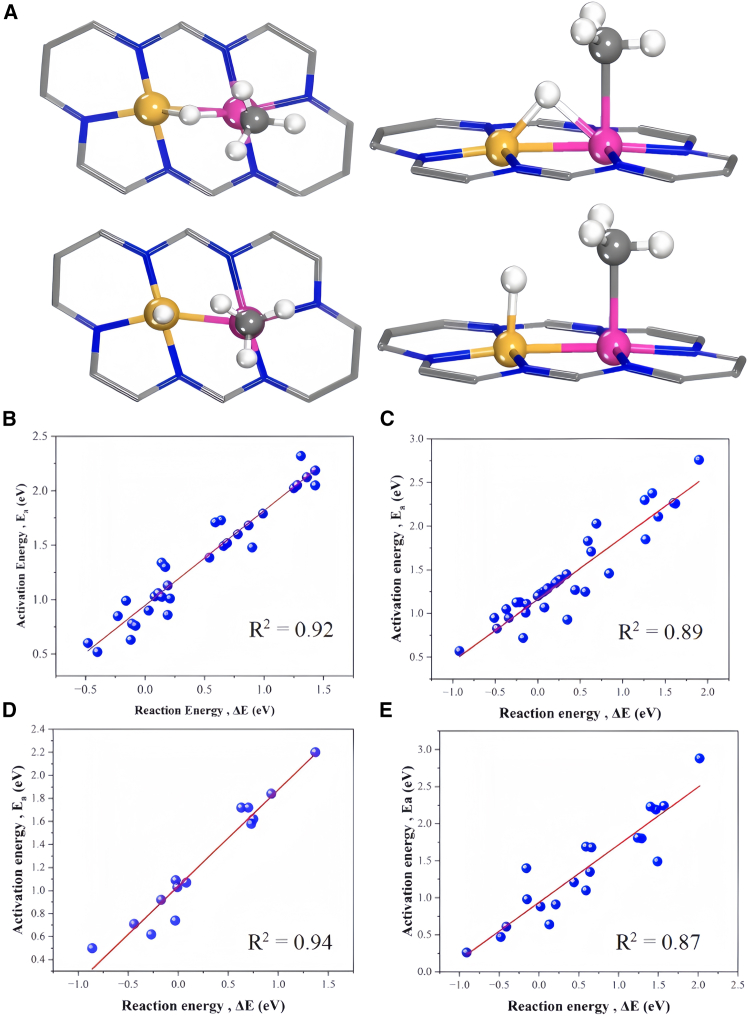
Typical decomposition configuratin and BEP relation (A) Methane decomposition configurations in different views, with the bridge site on the upper image and the single site on the lower image. The BEP relationship for the activation of the first C–H bond of (B) CH_4_, (C) C_2_H_6_, (D) C_3_H_8_ and primary C–H bond activation, and (E) C_3_H_8_ and secondary C–H bond activation*.*

The activation of C–H bonds in light alkane molecules is a key indicator for evaluating their catalytic performance. After C–H bond activation, there are two dissociated configurations based on the binding site of hydrogen as shown in Figure 2. Taking methane as an example, the abstracted hydrogen from methane molecule resulted from first C–H bond activation can bind either with two metal atoms as a bridge configuration (bridge site) or with a single metal atom (single site) as shown in Figure 2A. Moreover, C-H barrier associated bridge site is smaller than the counterpart of single site due to the more stabilized hydrogen atom as shown in Table S1.

**Figure 3:**
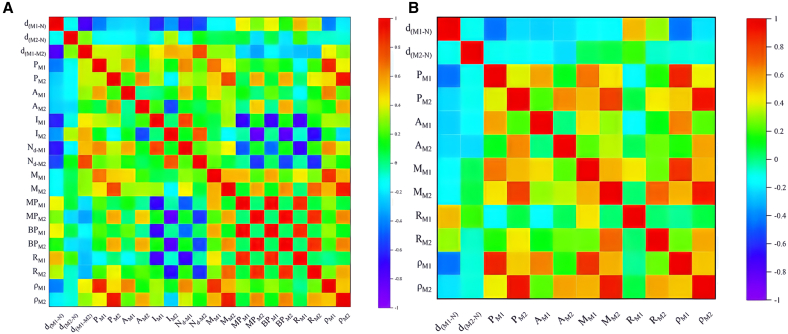
Feature analysis by Pearson method (A) Pearson correlation analysis of the initial features. (B) Pearson correlation analysis after screening.

As shown in Table S2, a bunch DACs is randomly selected for reaction barrier calculation. The calculations verified that BEP (Brønsted−Evans−Polanyi) relationship is valid for DACs regarding of C–H bond activation as shown in Figures 3B–3E as there is a good linear relationship between the calculated potential barrier and the reaction energy. In fact, BEP relation has been also reported to be applicable for other DACs.^25^ Therefore, BEP is applied to obtain the remaining DACs and complete C–H bond activation barrier.

### Feature engineering

To pin down the most important factors associated activity and interpret the outcome from ML. Based on the above DFT results, 21 features were selected to determine the relationship between the intrinsic characteristics of DACs and the activation of the first C–H bond of light alkane molecules. The first three descriptors provide the geometry of the DACs, while the remaining descriptors are related to the intrinsic properties of the metal atoms, and these descriptors are widely used as feature sets for training ML models.^26^ Although the above features covered a wide range of properties, they might be correlated with each other and become redundant. To gain more precise designation, a Pearson correlation coefficient matrix was used to identify the correlation between feature pairs as shown in Figure 3A, and high correlated features are removed which resulted in the left features shown in Figure 3B and Table S3. Moreover, the Pearson correlation coefficient heatmap in Figure 3B illustrates the weak linear correlation between these features, proving that each selected feature is independent and critical for training ML models. In particularly, the distance from the metal atom to the N atom (d_M1_/_M2-N_) was negatively correlated with the adsorption energy and energy barrier, reflecting an increase in adsorption energy and a decrease in energy barrier when the bond length was shortened.

### The performance of ML models

In most cases, both adsorption and C–H bond activation are crucial for the light alkane molecule activation. However, the previous single task ML methods did not predict both energy barriers and adsorption energies at same footing, which is not capable to give fair evaluation of catalytic performance.^20^ To circumvent this limitation, transformer and multilayer perceptrons models^27^ (MLP), which can perform multi-task learning, are chosen in current work, and a meaningful comparison between two methods is also carried out. It is noted that MLP is one of the most commonly used ML algorithms in multi-task learning, and its basic frameworks are feedforward neural networks.^28^ The comparison between two methods can provide more evidence for the appropriate model for screening of DACs.

The normalization and preprocess the raw data obtained from DFT calculations are executed first. We chose to study the portion of light alkane adsorption energy that accounts for a large proportion as shown Figure 2. This is because the adsorption energy data obtained through DFT calculation has a large span, which may pose a risk of overfitting, and randomly shuffling the data is also carried out to avoid potential bias. In addition, the training set and the test set are randomly divided into data according to the proportion of 70% and 30% to ensure objective evaluation. Transformer and MLP models were separately trained for 500 cycles.

**Figure 4:**
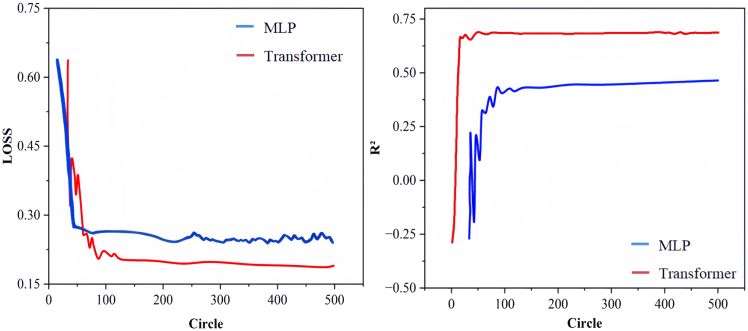
The LOSS and R^2^ values for MLP and transformer models for methane adsorption and activation on DACs

Using the optimized features, a preliminary comparison between transformer and MLP model is performed based on the data from methane adsorption and activation. For both adsorption and C–H bond activation, MLP model delivered a R^2^ score below 0.5, while it nearly reached 0.7 from transformer model which indicated that the latter is more capable to capture the essentials of light alkane molecule activation as shown in Table S4. The better performance of transformer model is further supported from analysis of loss function as shown in Figure 4, it can be seen that after 500 laps, the LOSS value of MLP stabilizes at around 0.25, and R^2^ stabilizes at around 0.47. On the other hand, LOSS value of the transformer is around 0.19, and R^2^ is around 0.70. The LOSS value of the transformer model tends to stabilize after 130 cycles, while R^2^ has a larger amplitude and an upward trend after 200 cycles. Therefore, the comparison indicated that transformer model is more suitable, and the model training cycles are further increased to 2000 cycles.

**Figure 5:**
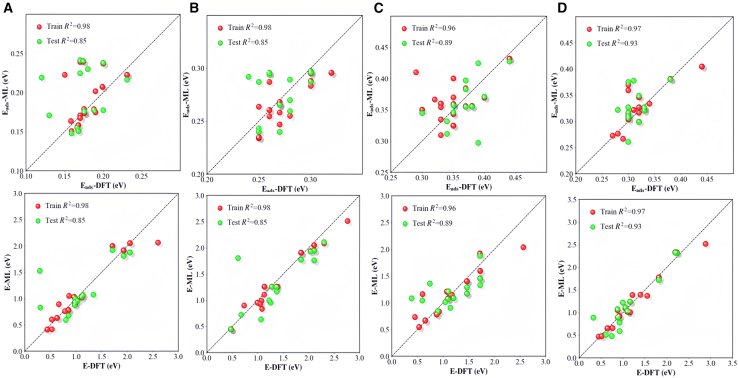
Transformer model predicts light alkane molecules adsorption and activation The first row represents adsorption energy, and the second row represents energy barrier. (A) CH_4_. (B) C_2_H_6_. (C) C_3_H_8_ via secondary hydrogen activation. (D) C_3_H_8_ via primary hydrogen activation.

The performance delivered by transformer model in this preliminary test validated its potential and reliability to predict alkane molecule activation which is deserved for further optimization. To this end, a decoder layer and an attention head were added to the existed model. The decoder layer gradually generates the output sequence through self-attention mechanism and encoder decoder attention mechanism. Self-attention ensures that the current generation position can focus on the generated part, while encoder decoder attention integrates the input information. The concatenation and linear transformation of multi head results enhance the model ability to express complex patterns. Moreover, dynamic model loading has been added to ensure support for multiple architectures and facilitate the expansion of new models. The input data are standardized and the dimensions are readjusted to adapt to the new model structure. Meanwhile, an adaptive output layer was designed to address the multi-output characteristics of predicting chemical adsorption energy and energy barrier. In addition, a dynamic validation strategy is adopted during the training process to monitor the performance of the model on the test set which can adjust the model weights in real-time on the data and save the best model through an early stopping mechanism. Finally, optimized model implements complete training log recording and visualization functions, which facilitates the analysis of the performance changes of the model at different stages. These improvements collectively enhance the practicality and interpretability of the model in adsorption energy and energy barrier prediction tasks. Also, the optimized model showed much improved capability to deal with the small size dataset compared with the frequently used ML models including adaptive boosting, KNN, RDF, and gradient boosting as shown in Figure S4. The comparison was executed by using the same dataset of methane adsorption and activation which clearly indicated that the tested ML methods showed a much inferior performance in terms of R^2^ compared with the current optimized transformer model. Moreover, the regression metrics (RMSE and R^2^) of optimized transformer model in terms of adsorption energy and C–H bond activation barrier of methane is significantly increased as shown in Figure 5A compared with preliminary test shown in Table S4. To further verify transformer model capability for light alkane molecule activation, the adsorption and activation of ethane and propane as cross-validation are also examined as shown in Figures 5B–5D which possessed R^2^ over 0.85. Therefore, the optimized transformer model is capable to deliver a good prediction for all tested light alkane molecules.

**Figure 6:**
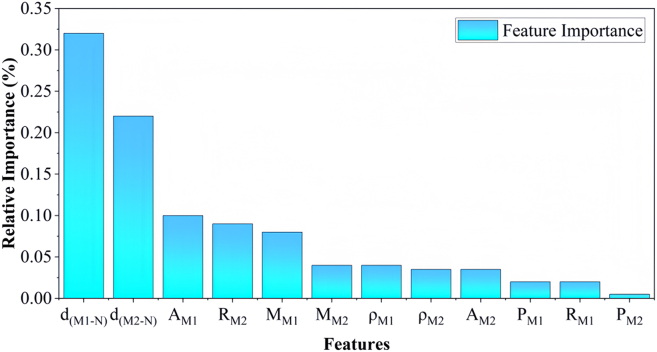
The importance regarding of light alkane molecules activaiton by using GBRT algorithm and the “final elimination” method

To gain more insight on the key feature influence on reactivity, the importance analysis was performed as shown in Figure 6 by using GBRT algorithm. For ranking the selected features, the GBRT based on the “last position elimination” algorithm revealed the features with the highest correlation. The most important factors for the activation of C–H bonds in light alkanes are the dM_1-N_ and dM_2-N_ which is consistent with the predictions of Pearson analysis.

### Conclusion

In summary, a joint first principles calculations and ML studies were conducted to predict the performance of DACs in the activation of light alkane molecule including methane, ethane, and propane, and regulate the design principle of DACs. DFT calculations indicated that BEP relation is valid for DACs regarding of C–H bond activation. Two multi-task ML models, transformer and MLP, were used to predict the adsorption energy and activation energy barriers of light alkane molecules, and transformer was found to outperform MLP. The fine-tuning transformer model further improved the performance with R^2^ exceeding 0.85 and extended to ethane and propane by using matrix multiplication and *Z* score, achieving synergistic prediction of adsorption energy and C–H bond activation energy barriers. Feature engineering indicated that the distance d_(M-N)_ between nitrogen atoms and metal center is a key descriptor affecting the catalytic activity of DACs for C–H bonds. Shortening the distance d_(M-N)_ can enhance the adsorption of light alkane molecules and reduce the activation energy barrier for C–H bonds. Overall, the current work provides an accurate and effective ML model to predict DACs reactivity for light alkane activation, and pave way for the further developments of DACs.

### Limitations of the study

Although this study reveals that progress has been made in the activation of light alkanes catalyzed by diatomic catalysts and the construction of ML prediction models, there are still some limitations: first, the support of DACs in the study is only nitrogen-doped graphene, and other common supports are not involved, and the metal combination is limited to 4/5 cycle transition metals, and is not included in rare earth metals, main group metals and other categories, leading to the universality of the research conclusion under different supports and metal systems to be verified; second, the DFT calculation and ML model only focused on the adsorption energy and C–H bond activation energy barrier of light alkanes, without considering the influence of dynamic reaction conditions such as temperature, pressure, reactant concentration on catalytic performance in the actual catalytic reaction, and did not involve key practical application indicators such as catalytic cycle stability and product selectivity, so the relevance with industrial catalytic scenarios needs to be further strengthened; In the future, it is necessary to combine other carrier metals and verify the model to fill these gaps.

## Resource availability

### Lead contact

Further requests for computational data, detailed methodologies, or collaborative inquiries should be directed to the lead contact, Bo Li (boli@synu.edu.cn).

### Materials availability

This study did not generate new physical materials.

### Data and code availability

• The code of this research framework has been uploaded to GitHub.https://github.com/BairanWang/Multi-task-machine-learning-to-predict-hydrocarbon-bond-activation-properties-of-low-carbon-alkanes
• See Data S1 for specific methods of ML. All other data reported in this paper will be shared by the lead contact upon request.
• Any additional information required to reanalyze the data reported in this paper are available from the lead contact upon request.

## Acknowledgments

This work was supported by 10.13039/501100001809National Natural Science Foundation of China (grant nos. 22372105 and 22172100); Basic Research Project of Education Office of Liaoning Province (JYTZD2023183); the Fundamental Research Funds for the Liaoning Universities (LJ212410166043); 10.13039/501100012145Shenyang Normal University (BS202208); and the Program for Excellent Talents in 10.13039/501100012145Shenyang Normal University.

## Author contributions

B.W. conceived the study, developed the methodology, performed formal analysis and investigations, conducted the calculations and simulations, curated the data, wrote the original draft, and created visualizations; X.S. and Z.Z. coordinated and supervised the research direction; W.X., L.S., and J.Q. contributed to validation and manuscript review; B.L. provided supervision, secured project resources and funding, and participated in critical revisions of the manuscript. All authors discussed the results and contributed to the final version of the paper.

## Declaration of interests

The authors declare no competing interests.

## STAR★Methods

### Key resources table

**Table:** 

REAGENT or RESOURCE	SOURCE	IDENTIFIER
**Software and algorithms**		

VASP	VASP Software GmbH	https://www.vasp.at
VASTA software package	JP-Minerals	https://jp-minerals.org/vesta/en/
Jmol	Open-source community	https://jmol.sourceforge.net/
p4vasp	VASP post-processing tool	https://p4vasp.at

### Method details

#### DFT calculations

All calculations are done using the Vienna ab initio simulation package. For valence electrons, a plane wave basis set is used with an energy cutoff of 400 eV. The modified Perdew Burke Ernzerhof (PBE) functional is used as an exchange correlation functional approximation which is known for description of the adsorption^29^ and energy barrier on transition metal surfaces. The thickness of the vacuum is 12 Å. DFT-D3 method was used for van der Waals correction.^30^ The total energy convergence is of 1 × 10^−6^ eV and a force tolerance of 0.03 eV/Å is used in all structure optimizations. To evaluate the stability of the doped catalyst, the formation energy can be calculated using formula:

(Equation 1) $$E_{f}=\frac{1}{n}\left(E_{DACs}-n_{c}\mu_{c}-n_{N}\mu_{N}-\mu_{M1}-\mu_{M2}\right)$$

Among them E_DACs_, μ_C_, μ_N_, μ_M1_, μ_M2_ represent the total energy of DACs, the chemical potential of carbon, nitrogen, and metal species respectively. The chemical potential of carbon, nitrogen, and _metal_ species is referred to the graphene, nitrogen molecule, and bulk metal respectively. n represented the number of atoms.

#### The adsorption energy E_ads_ was calculated as

(Equation 2) $$E_{ads}=E_{adsorbateslab}-\left(E_{slab}+E_{adsorbate}\right)$$

where E_(adsorbate/slab)_ is the total energy of adsorbate and slab; E_slab_ and E_adsorbate_ are the energy of the slab and the adsorbate (isolated CH_4_/C_2_H_6_/C_3_H_8_ molecules) respectively. The reaction _pathways_ and energy barriers were calculated by using the climbing nudged elastic band (CI-NEB) method and transition state is identified as only one imagery frequency from vibration analysis.^31^ The reaction barrier was calculated as the difference between the initial state and transition state on the pathway and the reaction energy was calculated as the difference between initial state and final state on pathway.

(Equation 3) $$E_{a}=E_{FS}-E_{IS}$$

(Equation 4) $$\Delta E=E_{FS}-E_{IS}$$

The E_FS_ and E_IS_ are the total energies of the final state (FS) and the initial state (IS) respectively.

The bimetallic (M_1_, M_2_) candidates include Sn, Ti, V, Mn, Co, Fe, Ni, Cu, Zn, Y, Zr, Nb, Mo, Tc, Ru, Rh, Pd, Ag, Cd, Pt and Au which resulted in a series combination over 220 DACs. In particularly, some of metal candidates have been reported to have good performance in C-H bond activation of light alkane molecule.^32^ Furthermore, the nitrogen doped graphene is chosen as substrate for DACs as shown in Figure S1. It is noted the nitrogen configuration in Figure S1 is widely explored for both SACs and DACs, and is verified from experimental synthesis.

#### ML method

**Figure 7:**
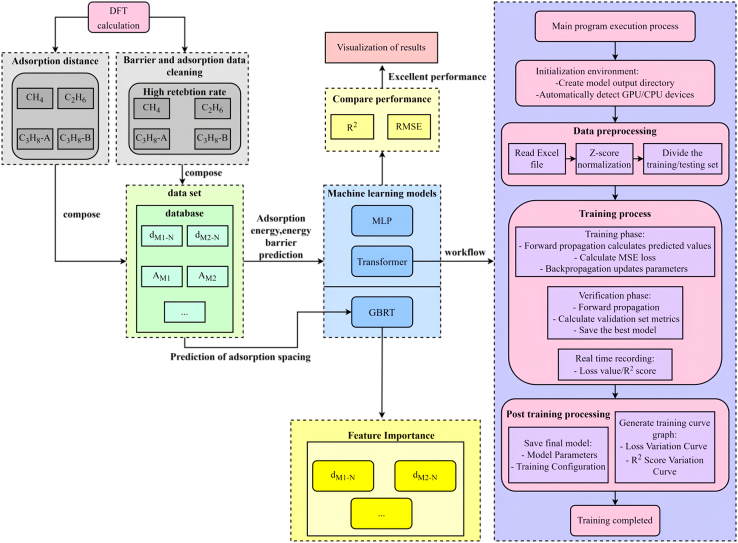
Workflow diagram of first principles calculations and ML For propane molecule, both primary (C_3_H_8_-A) and secondary (C_3_H_8_-B) C-H bond activation are explored.

Recently, Transformer model has been applied to tackle intractable chemistry issues due to excellent sequence modeling capabilities and parallelization advantages.^33^ In particularly, the multi-head attention mechanism used in this model can effectively analyze the long-range correlation characteristics in chemical data, and significantly improve the accuracy in molecular property prediction tasks. By leveraging this property of Transformer model, our work developed a framework for predicting the performance of DACs regarding C-H bond activation in light alkane molecule as illustrated in Figure 7. The system includes four functional modules: data preprocessing, model architecture design, training optimization, and result visualization, achieving an end-to-end computational analysis from raw data to performance prediction. More details about the setup of ML are included in SI.

### Quantification and statistical analysis

The computational analyses in this study were performed using several specialized software packages. Density functional theory (DFT) calculations were performed using the Vienna Ab Initio Simulation Package (VASP), implementing strict convergence criteria of 1 × 10^−6^ eV for energy and 0.03 eV/Å for forces. VASP facilitated structural optimizations, electronic structure analyses, and transition state searches through CI-NEB and dimer methods. Structural visualization and charge density distributions were generated using VESTA and Jmol (Figures 2A; S1 and S3). By using Origin Software for fitting, summarizing and feature screening (Figures 1, 2B, 2C, 3; S2; S5). Use Python for machine learning calculation and get fitting results (Figures 4, 5, 6, and 7; S4).

### Additional resources

All data generated during this computational study are included in the manuscript and supplementary information. No external resources were required.

## Supplemental information

Document S1. Figures S1–S5 and Tables S1–S5

Data S1. Machine learning method, related to STAR Methods
