# Supplementary material for: First-principles calculations steered multi-task transformer model to screen dual-atom catalysts for C-H activation
Source: iScience. 2025 Nov 21;28(12):114182. doi: 10.1016/j.isci.2025.114182 (PMC12719781; doi:10.1016/j.isci.2025.114182)
Supplement: Document S1. Figures S1–S5 and Tables S1–S5 [file mmc1.pdf]

iScience, Volume *28*

Supplemental information

First-principles calculations steered multi-task transformer model to screen dual-atom catalysts for C-H activation

BaiRan Wang, WeiHang Xu, XiaoYing Sun, Qi Ji, LingLing Shang, Zhen Zhao, and Bo Li

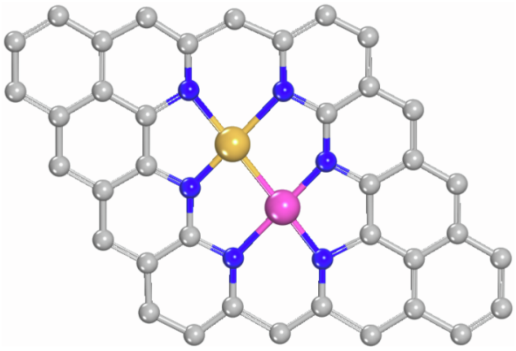

Figure S1. Illustration of DACs configuration considered in current work. The metal species are bonded with six nitrogen atoms on graphene substrate. Color code: light gray is carbon, blue is nitrogen, and yellow, pink are metal atoms

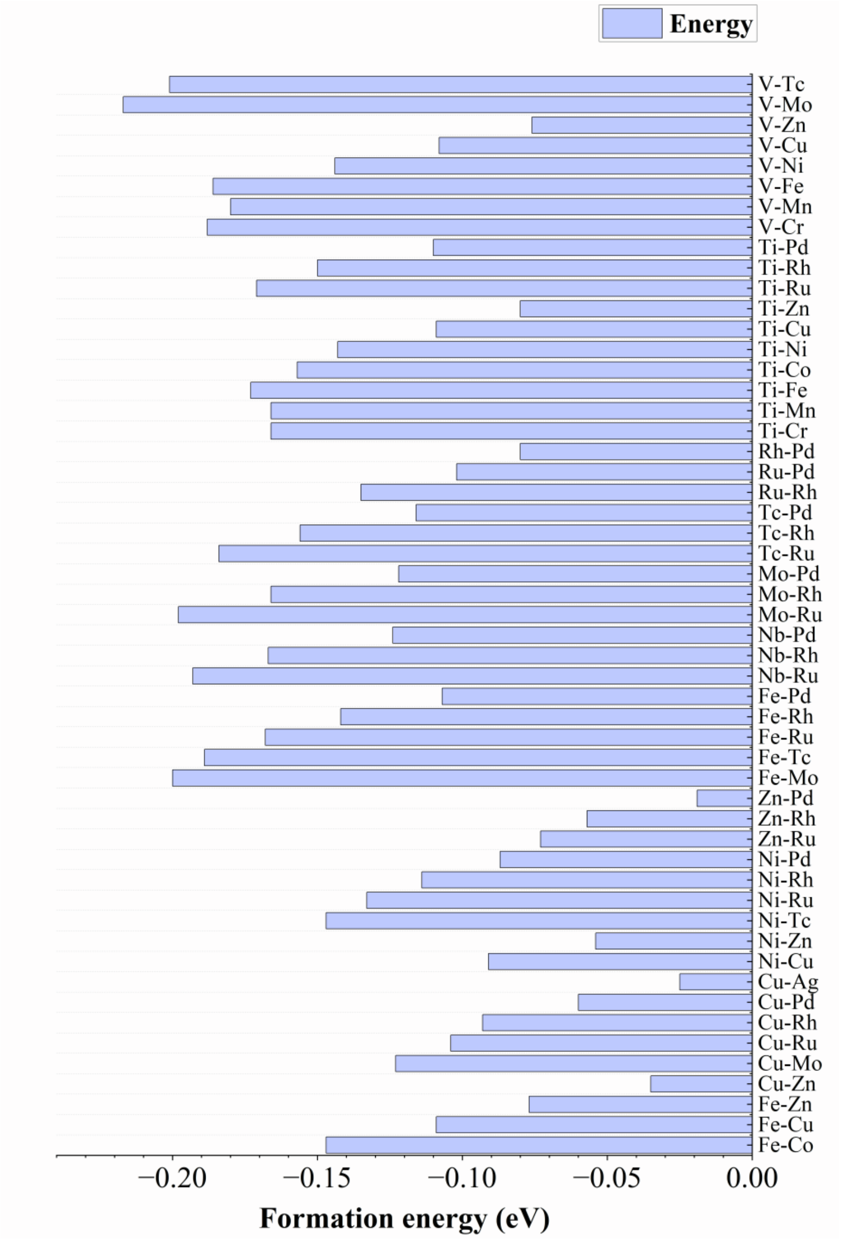

Figure S2. Calculated formation energy of DACs

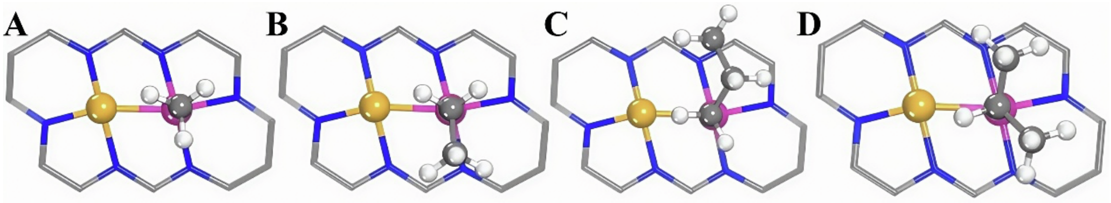

Figure S3. The configurations of adsorbed alkane molecules. A. methane, B. ethane, C. propane via primary hydrogen, D. propane via secondary hydrogen.

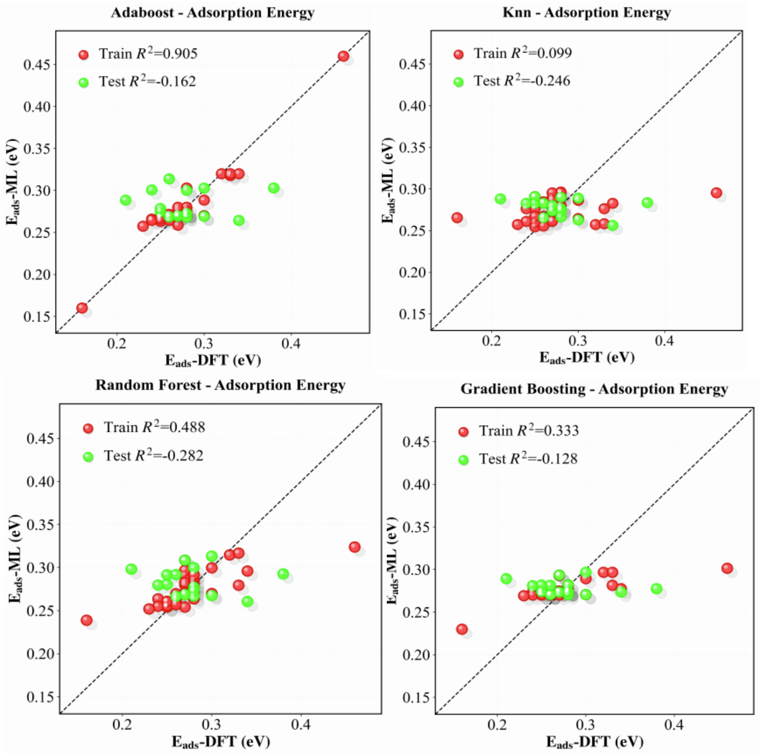

Figure S4. The regression performance of Adaboost, KNN, RDF, GBR based on methane adsorption and activation.

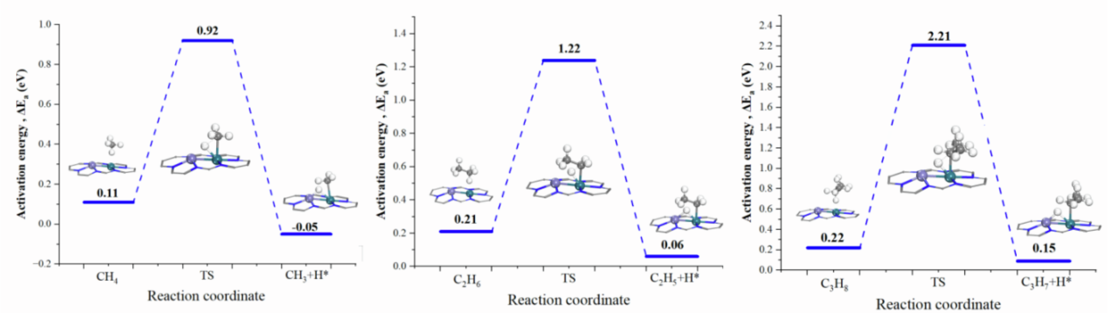

Figure S5. Representative configurations of initial, transition state, and final state on potential energy profile for first C-H bond activation in methane, ethane, and propane (from left to right).

Table S1. The average of C-H bond energy barrier and reaction energy at the bridge and single site respectively.

Site	Energy barrier (eV)
Single site	1.19
Bridge site	1.03

Table S2. Randomly selected DACs for reaction and barrier calculations including both bridge and single configuations

	Methane to Methyl	Methane to Methyl
**Promoter element**	**Reaction Energy(eV)**	**Energy Barrier(eV)**
FeCo-Fe	-0.19	0.63
FeCo-Co	-0.20	1.32
FeCu-Fe	-0.18	1.93
FeCu-Cu	-0.17	1.31
FeZn-Fe	-0.23	0.85
FeZn-Zn	-0.24	0.99
RhPd-Rh	-0.16	1.07
RhPd-Pd	-0.13	1.74
NiCu-Ni	-0.16	0.97
NiCu-Cu	-0.16	2.21
CuRh-Cu	-0.17	2.05
CuRh-Rh	-0.16	1.73
CuPd-Cu	-0.17	0.98
CuPd-Pd	-0.15	2.46
NiRh-Ni	-0.16	1.03
NiRh-Rh	-0.16	1.48
NiZn-Ni	-0.19	1.71
NiZn-Zn	-0.18	2.32
NiTc-Ni	-0.17	0.60
NiTc-Tc	-0.18	0.93
	**Ethane to Ethyl**	**Ethane to Ethyl**
**Promoter element**	**Reaction Energy(eV)**	**Energy Barrier(eV)**
FeCo-Fe	-0.26	0.47
FeCo-Co	-0.28	0.55
FeCu-Fe	-0.27	1.85
FeCu-Cu	-0.27	0.93
FeZn-Fe	-0.30	0.47
FeZn-Zn	-0.33	1.01

ZnRu-Zn	-0.44	1.13
ZnRu-Ru	-0.37	0.95
CuMo-Cu	-0.25	1.13
CuMo-Mo	-0.26	0.57
CuRh-Cu	-0.25	2.11
CuRh-Rh	-0.22	1.71
CuZn-Cu	-0.27	2.76
CuZn-Zn	-0.28	2.27
NiRh-Ni	-0.26	1.07
NiRh-Rh	-0.26	1.46
NiZn-Ni	-0.30	2.03
NiZn-Zn	-0.27	2.30
NiTc-Ni	-0.23	1.03
NiTc-Tc	-0.30	0.83
	**Propane para hydrogen to Propyl**	**Propane para hydrogen to Propyl**
**Promoter element**	**Reaction Energy(eV)**	**Energy Barrier(eV)**
FeCo-Fe	-0.35	0.65
FeCo-Co	-0.36	0.74
FeZn-Fe	-0.44	1.09
FeZn-Zn	-0.42	1.03
CuRu-Cu	-0.37	0.62
CuRu-Ru	-0.33	1.62
CuMo-Cu	-0.35	0.92
CuMo-Mo	-0.33	0.50
CuRh-Cu	-0.38	1.72
CuRh-Rh	-0.31	1.71
NiRh-Ni	-0.33	1.07
NiRh-Rh	-0.34	1.84
NiZn-Ni	-0.35	1.72
NiZn-Zn	-0.37	2.20
NiTc-Ni	-0.34	0.71
NiTc-Tc	-0.36	0.78
NiRu-Ni	-0.35	0.60
NiRu-Ru	-0.31	1.58
	**Propane primary hydrogen to Propyl**	**Propane primary hydrogen to Propyl**
**Promoter element**	**Reaction Energy(eV)**	**Energy Barrier(eV)**
FeCo-Fe	0.21	0.91
FeCo-Co	0.13	0.64
FeZn-Fe	0.59	1.1
FeZn-Zn	1.29	1.8
CuRu-Cu	0.02	0.88

CuRu-Ru	-0.15	0.98
CuMo-Cu	2.02	2.88
CuMo-Mo	1.57	2.24
CuRh-Cu	-0.16	1.4
CuRh-Rh	1.24	1.81
NiRh-Ni	0.59	1.69
NiRh-Rh	1.47	2.19
NiZn-Ni	1.49	1.49
NiZn-Zn	1.4	2.23
NiTc-Ni	0.66	1.68
NiTc-Tc	-0.48	0.47
NiRu-Ni	-0.91	0.26
NiRu-Ru	0.64	1.35

Table S3. The symbol and meaning of 21 selected features

**Feature**	**Meaning**
$d_{(M1-N)}$	M_1 atom to N distance
$d_{(M2-N)}$	M_2 atom to N distance
$d_{(M1-M2)}$	The distance between M_1 atom and M_2 atom
P_{M1}	Electronegativity of M_1 atoms
P_{M2}	Electronegativity of M_2 atoms
A_{M1}	Electron affinity of M_1 atoms
A_{M2}	Electron affinity of M_2 atoms
I_{M1}	The first ionization energy of M_1 atoms
I_{M2}	The first ionization energy of M_2 atoms
N_{d-M1}	The number of d electrons in M_1 atoms
N_{d-M2}	The number of d electrons in M_2 atoms
M_{M1}	The mass of M_1 atoms
M_{M2}	The mass of M_2 atoms
MP_{M1}	Melting point of M_1 atoms
MP_{M2}	Melting point of M_2 atoms
BP_{M1}	Boiling point of M_1 atoms
BP_{M2}	Boiling point of M_2 atoms
R_{M1}	The radius of M_1 atoms
R_{M2}	The radius of M_2 atoms
ρ_{M1}	Density of M_1 atoms
ρ_{M2}	Density of M_2 atoms

Table S4. Performance evaluation of MLP and Transformer test sets

**Machine learning models**	MLP	Transformer
	Testing set R^2	Testing set R^2
**Adsorption energy**	0.47	0.70
**Energy barrier**	0.47	0.70

Table S5. The hyperparamter setting of improved Transformer model

**Parameter name**	**Command line settings**	**Variable name in code**	**Explain**
--num_layers	5	args.num_layers	Encoder layers
--hidden_size	128	args.hidden_size	Hidden layer dimension
--lr	1e-4	args.lr	Adam optimizer learning rate
--epoch	2000	args.epochs	Number of training rounds
--seed	42	args.seed	Random seed
