# Supplementary material for: First-principles calculations steered multi-task transformer model to screen dual-atom catalysts for C-H activation
Source: iScience. 2025 Nov 21;28(12):114182. doi: 10.1016/j.isci.2025.114182 (PMC12719781; doi:10.1016/j.isci.2025.114182)
Supplement: Data S1. Machine learning method, related to STAR Methods [file mmc2.pdf]

Data S1. Machine learning method, related to STAR Methods.

In the data preprocessing stage, the adsorption energy and energy barrier of low-carbon alkanes calculated by DFT are cleaned, and the larger proportion is retained and combined with the selected features to form a dataset. The adsorption distance of low-carbon alkanes is combined with the selected features to form another dataset. Therefore, the two machine learning algorithms have the same underlying logic and can have the same processing mode when compared. In terms of training optimization, the R^2 and RMSE loss values of the two algorithms were compared successively. The higher the R^2 value, the higher the fitting effect of the model, and the lower the RMSE loss value, the more accurate the model can predict. After selecting a model with high fitting degree and low loss value, visualizing the results can more directly show the prediction effect, and predict the results based on the obtained images.

The key performance indicators of C-H bond activation are the adsorption energy and barrier of C-H bond breaking which is used to form a multi-task learning framework. The dataset adopts a hierarchical random partitioning strategy, generating training and validation sets in a 7:3 ratio, and adapting them to the input specifications of the deep learning model through tensor reshaping (samples × features × channels). All architectures adopt a deep and scalable design, with a default configuration of 5 hidden layers (128 neurons per layer), and the output layer uses a linear activation function to achieve four-dimensional performance prediction. Specifically, the model achieves multi-task learning through parameter sharing mechanism, automatically balancing the gradient update strength of different performance indicators during backpropagation. Under this framework, it establishes an end-to-end multi-task learning model for Diatomic catalyst, breaking through the dimensional limitations of traditional descriptor methods such as Material descriptor method, molecular descriptor method which has stimulated the potential for high-throughput catalyst design capabilities.
